# Interaction of miR-200a-3p with YAP regulates cell proliferation and metastasis differentially in HPV-positive and HPV-negative cervical cancer cells

**DOI:** 10.1186/s12885-022-10118-0

**Published:** 2022-10-04

**Authors:** Hong Chen, Lingling Gu, Min Zhang, Huifen Chen, Hong Liao, Xueping Cao, Lu Yu, Jun Zhang

**Affiliations:** 1 Department of Clinical Laboratory, Shanghai Key Laboratory of Maternal Fetal Medicine, Shanghai Institute of Maternal-Fetal Medicine and Gynecologic Oncology, Shanghai First Maternity and Infant Hospital, School of Medicine, Tongji University, Shanghai, 200092 China; 2 Key Laboratory of Brain Functional Genomics (ECNU), Ministry of Education, School of Life Sciences, East China Normal University, Shanghai, 200062 China; 3 Department of Clinical Laboratory, Shanghai Public Health Clinical Center, Fudan University, Shanghai, 201508 China; 4 Comprehensive Department of Traditional Chinese Medicine, Putuo Hospital, Shanghai University of Traditional Chinese Medicine, Shanghai, 200062 China

**Keywords:** miR-200a-3p, YAP; HPV, Cervical cancer

## Abstract

**Background:**

Although evidence has revealed that miR-200a-3p is involved in the malignant progression of various tumors, the regulatory mechanism of miR-200a-3p in the development of cervical cancer (CC) cells with different HPV statuses remains unknown. The present study was to investigate the differential effects of either miR-200a-3p or YAP on tumorous cells’ fate in vitro in HPV-negative and HPV-positive cervical cancer cell models, and to explore if the changes in proliferation, migration, and invasion of the CC cells with different HPV statuses could be attributed to the differential interactions between miR-200a-3p and YAP.

**Methods:**

The colony formation assays, EDU assays and Transwell assays were performed for CC cell proliferation, migration and invasion capacities analysis. The prediction of downstream targets of miR-200a-3p was performed by bioinformatical databases. The dual-luciferase reporter assays were used to validate the binding sites of miR-200a-3p and YAP. The qRT-PCR assays were performed to quantify the mRNA expression of miR-200a-3p and YAP, and the protein levels of YAP were examined by Western blot analysis.

**Results:**

The results demonstrated that miR-200a-3p overexpression suppressed proliferation, migration, and invasion of the HPV-negative C33A cells but promoted the growth and metastasis of HPV-positive CC cells, while YAP promoted the cell growth and metastasis not only in HPV-negative but also in the HPV-positive CC cells. The suppressive role of miR-200a-3p in C33A cells appeared to be mediated partially by direct interaction with YAP, and YAP might participate in miR-200a-3p-mediated cellular changes in CC cells differing from not only the presence or absence of HPV but even also the subtypes of HPV of CC cells. Meanwhile, we preliminarily revealed that the expression level of miR-200a-3p was significantly decreased in HPV-negative, but not in HPV16-positive cervical neoplasm mucus samples.

**Conclusion:**

miR-200a-3p-mediated functional changes of YAP exhibited regulatory effects on cells’ fate differentially in HPV-negative and HPV-positive cervical cancer cells.

**Supplementary Information:**

The online version contains supplementary material available at 10.1186/s12885-022-10118-0.

## Introduction

Although persistent high-risk human papillomaviruses infection is the main causative agent of cervical cancer(CC) [1, 2], previous studies also reported that a part of CC patients was consistently HPV-negative [3], suggesting that, except for HPV infection, more essential factors should also be involved in the occurrence and development of cervical cancer.

Recently, the incidence and death rate of CC have reduced due to HPV screenings and HPV vaccination being applied in clinical practice [4]. However, a considerable number of CC patients still die from distant metastasis and recurrence [5]. It is thus important to find out novel molecular mechanisms that may better explain the pathogenesis of the cancer.

Evidence has emerged that microRNAs are involved in the pathogenesis and progression of various cancers [6]. MiR-200a-3p, belonging to the miR-200 family, has been shown to be implicated significantly in pathological changes of several common cancers due to its critical oncogenic or tumor-suppressive functions in modulation of cadherin superfamily or Hippo signaling pathway [7–10]. However, roles of miR-200a-3p in CC are seldom reported, especially its specific molecular mechanisms underlying its functional responsibilities to HPV-positive and HPV-negative CC cells still remain unclear.

YAP (Yes-associated protein) is the main component of the Hippo signaling pathway and mainly functions as an oncogene in various cancers [11, 12]. Up-regulation of YAP by Hsa_circ_0005273 could promote breast cancer progression [13]. Previous studies have shown an intimate relationship between YAP with regulatory functions of miRNAs [14]. For example, the interaction between YAP and miR-27b-3p could inhibit the proliferation of breast cancer cells [15]. Inhibition of the miR-381-YAP-Snail axis by Metformin could disrupt the growth and metastasis of NSCLC [16]. Moreover, a higher expression level of YAP was observed in CC tissues that contributed to the proliferation and migration of CC cells [17]. Nevertheless, the relationship between miR-200a-3p and YAP in CC development is still unclear, and especially whether miR-200a-3p could and how to differentially regulate YAP in response to the cellular and molecular changes in HPV-positive or HPV-negative CC cells need to be clarified. This study was thus conducted to investigate the differential effects of either miR-200a-3p or YAP on tumorous cells’ fate in vitro in HPV-negative and HPV-positive CC cell models, and then to explore if the changes in proliferation, migration and invasion of CC cells under different HPV statuses could be attributed to the differential molecular interactions between miR-200a-3p and YAP, which might give insights with clinical importance into a rational explanation about the progression and metastasis of HPV-negative cervical cancer.

## Materials and methods

### Patients and samples

HPV16-positive (*n* = 14), HPV-negative (*n* = 14) cervical neoplasm mucus samples and normal samples (*n* = 14) were collected by gynecologists of the Shanghai First Maternity and Infant Hospital according to clinical standard protocols. Briefly, the cervical brush was placed on the cervix and rotated 5 times clockwise. The brush was then put into a vial containing the cell preservation solution (Hybribio). The sample was stored at 4 °C for less than 48 h for HPV genotype test. For HPV positivity and its genotype, the mucus DNA was extracted using the Fully Automated Nucleic Acid Extraction System AutoPrep96 HBNP-9600A (Hybribio, China) which is based on magnetic bead separation technology. The PCR amplification and the hybridization between amplified DNA amplicons and type-specific HPV probes located inside the “Hybrimem” were performed by using the 21 HPV GenoArray Diagnostic Kit according to the manufacturer’s protocols (Hybribio, China). The HPV positive or negative was determined by the HPV hybridization spot with or without bluish-violet dots accordingly. The specific HPV genotype of the positive spot was identified by referring to the HPV typing distribution map of the membrane strip (Hybribio, China). After the genotyping test, the remaining samples were stored at -80 °C for further use. This study was approved by the Ethics Committee of Shanghai First Maternity and Infant Hospital (No. KS21150), and the informed consent was obtained from all subjects.

### Cell lines and transfection

The human CC cell lines (HPV-negative C33A, HPV16-positive Siha, HPV18-positive Hela) were purchased from American Type Culture Collection (ATCC), and cultured in DMEM high glucose medium complemented with 10% fetal bovine serum and 1% penicillin–streptomycin at 37℃ containing 5% CO_2_. The miR-200a-3p mimics/miR-NC were obtained from Ribobio (Guangzhou, China). Sh-miR-200a-3p/sh-NC and pGMLV-YAP plasmid/Vector were purchased from Genomeditech (Shanghai, China). Cells were transfected using Lipofectamine 3000 (Invitrogen).

### Quantitative real-time PCR

Total RNA of CC cells was extracted using TRIZOL (Invitrogen). Cervical mucus miRNA was extracted using miRcute Serum/Plasma miRNA Isolation Kit (TIANGEN), and cel-miR-39-3p was used as an internal control for miRNA. The reversed transcription of RNA and qPCR was performed with Primer Script RT Reagent Kit and SYBR Green Master Mix kit (Takara). Reverse transcription for miRNAs was implemented with a Primer Script RT reagent Kit (Takara). qRT-PCR assay of mucus miRNA was performed by using Primer Script RT Reagent Kit and SYBR Green Master Mix kit (Takara). Bulge-loop miR-200a-3p/U6 qRT-PCR Primer Sets were designed by Ribobio (Guangzhou, China). GAPDH and U6 were used as the internal control for mRNA and miRNA, respectively. Relative RNA levels were calculated using the 2^−△△CT^ method. The primers were listed as follows:

**Table:** 

GAPDH:	Forward, 5’-CGCTGAGTACGTCGTGGAGTC-3’
	Reverse, 5’-GCTGATGATCTTGAGGCTGTTGTC-3’
YAP:	Forward, 5’-TAGCCCTGCGTAGCCAGTTA-3’
	Reverse, 5’-TCATGCTTAGTCCACTGTCTGT-3’

### Colony formation assay

Hela, Siha, and C33A cells were placed into 6-well plates with 800 ~ 1000 cells per well and transfected with miR-NC/miR-200a-3p mimics, pGMLV-YAP plasmid/Empty Vector, respectively. After incubation at 37 °C for 2 weeks, 4% paraformaldehyde was used to fix for 20 min, then 6-well plates were washed with 1xPBS three times and fixed with 0.1% crystal violet solution for 20 min. All colonies were photographed and counted.

### EDU assay

Transfected cells were seeded in 24-well plates at a density of 3 × 10^4^ cells overnight. The assay was performed with EDU Cell Proliferation Kit with Alexa Fluor 555 (CellorLab). After incubated with 10 ul of EDU solution for 2 h at 37 °C, the cells were washed and stained following the manufacturer’s instructions. Representative images were acquired by a Leica microscope with 100 × magnification. The EDU-positive cells were calculated by the following formula: EDU-positive rate = EDU-positive cell count/ (EDU-positive cell count + EDU-negative cell count) × 100%.

### Transwell assay

Chambers (8-μm-pore size, Corning) and Matrigel (Corning) were used for cell invasion assays and chambers without Matrigel were used for migration assays. 1.2 × 10^5^ cells/per chamber were seeded into the upper chamber with serum-free medium and incubated in the lower chamber with 10% FBS complete culture medium for 24 h for migration and 48 h for invasion. The cells were fixed with 4% paraformaldehyde and stained with crystal violet solution. The representative images were acquired by a Leica microscope with 100 × magnification.

### Western blot

Cells were lysed in RIPA Lysis Buffer (Beyotime) with a proteinase inhibitor cocktail (EpiZyme). The lysed protein was mixed with 5 × loading buffer (EpiZyme), separated in 10% SDS-PAGE (Beyotime, and transferred onto PVDF membranes (0.45 um, Millipore). Membranes were masked with QuickBlock™ Blocking Buffer (Beyotime) for 2 h. The blots were properly cut according to target protein sites into several parts prior to hybridization with primary antibodies, and every blot was then incubated with their primary antibody correspondingly: β-actin (4970S,1:1000, CST) and YAP (14074 T,1:1000, CST) overnight at 4 °C. After washing, the membranes were probed with the secondary Anti-rabbit IgG, HRP-linked Antibody (7074S, 1:2000, CST) for 2 h at room temperature. The protein bands were visualized and evaluated with Tanon Imaging System and ImageJ software, respectively. β-actin was used as an internal control. All full-length Western blot images are shown in Additional File 2.

### Luciferase reporter assay

Prediction of potential genes targeted by miR-200a-3p was performed by using internet databases including miRWalk (http://mirwalk.umm.uni-heidelberg.de), miRNet (https://www.mirnet.ca/miRNet/home.xhtml), miRDB (http://mirdb.org/download.html), Targetscan (http://www.targetscan.org/vert_72/) and miRTarBase (data was presented in Additional file 3). The data from Starbase 3.0 (https://starbase.sysu.edu.cn/) showed a negative correlation between YAP and miR-200a-3p in cervical neoplasm tissues. The dual reporter plasmids H_YAP 3’-UTR WT and YAP 3’-UTR Mut were used for confirmation of the binding site of miR-200a-3p and YAP. Cells were cultured in 24-well plates (5 × 10^4^ cells/well) and co-transfected with miR-200a-3p mimics/miR-NC and WT/Mut H_YAP1 3’-UTR in Lipofectamine 3000 (Invitr-ogen), respectively. Luciferase activities were measured by the Dual-Luciferase Reporter Assay Kit (Genomeditech) according to the manufacturer’s protocols.

### Statistical analysis

Each experiment was repeated at least three times independently. Statistical analysis of two groups was performed by using unpaired Student’s t-tests. Data were presented as mean ± SD and were analyzed by GraphPad Prism 8.0 (USA). Statistical significance was indicated as follows: *p < 0.05, **p < 0.01, ***p < 0.001, ns, not significant.

## Results

### Up-regulation of miR-200a-3p plays differential roles in HPV-negative and HPV-positive CC cells

**Fig. 1:**
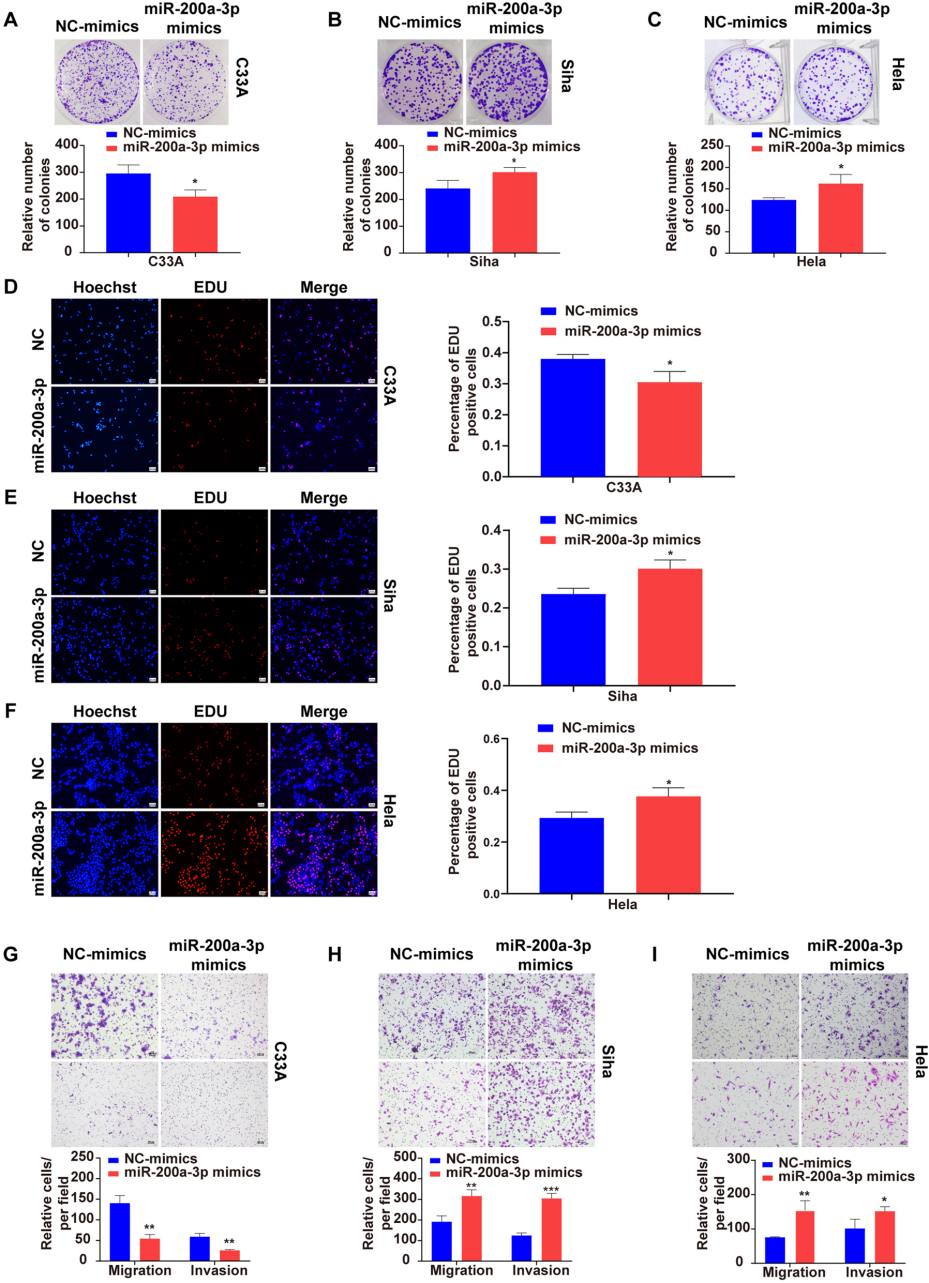
Differential effects of miR-200a-3p overexpression on proliferation and invasion of HPV-negative and HPV-positive CC cells***.*** Colony forming was inhibited in C33A **(A)**, and promoted in Siha **(B)** and Hela cells **(C)**. EDU-positive cells were decreased in C33A **(D)**, and increased in Siha **(E)** and Hela cells **(F)**. Cell migration and invasion were declined in C33A **(G)**, and enhanced in Siha **(H)** and Hela cells **(I)**, respectively. 100 × Magnification. *p < 0.05, **p < 0.01, ***p < 0.001, ns: not significant

With transfections of three cell lines with miR-200a-3p mimics/miR-NC (Supplementary Fig. S1 A in Additional file 1), the colony formation and EDU assays were performed and showed that miR-200a-3p overexpression suppressed the proliferation of HPV-negative C33A cells (Fig. 1A, D), but promoted growth of HPV16-positive Siha (Fig. 1B, E) and HPV18-positive Hela cells (Fig. 1C, F). The transwell assay showed that miR-200a-3p suppressed migration and invasion of C33A cells (Fig. 1G), but enhanced migration and invasion capacity in Siha and Hela cells (Fig. 1H, I).

### Effects of miR-200a-3p on function of YAP are differentially related to HPV statuses in the CC cells

**Fig. 2:**
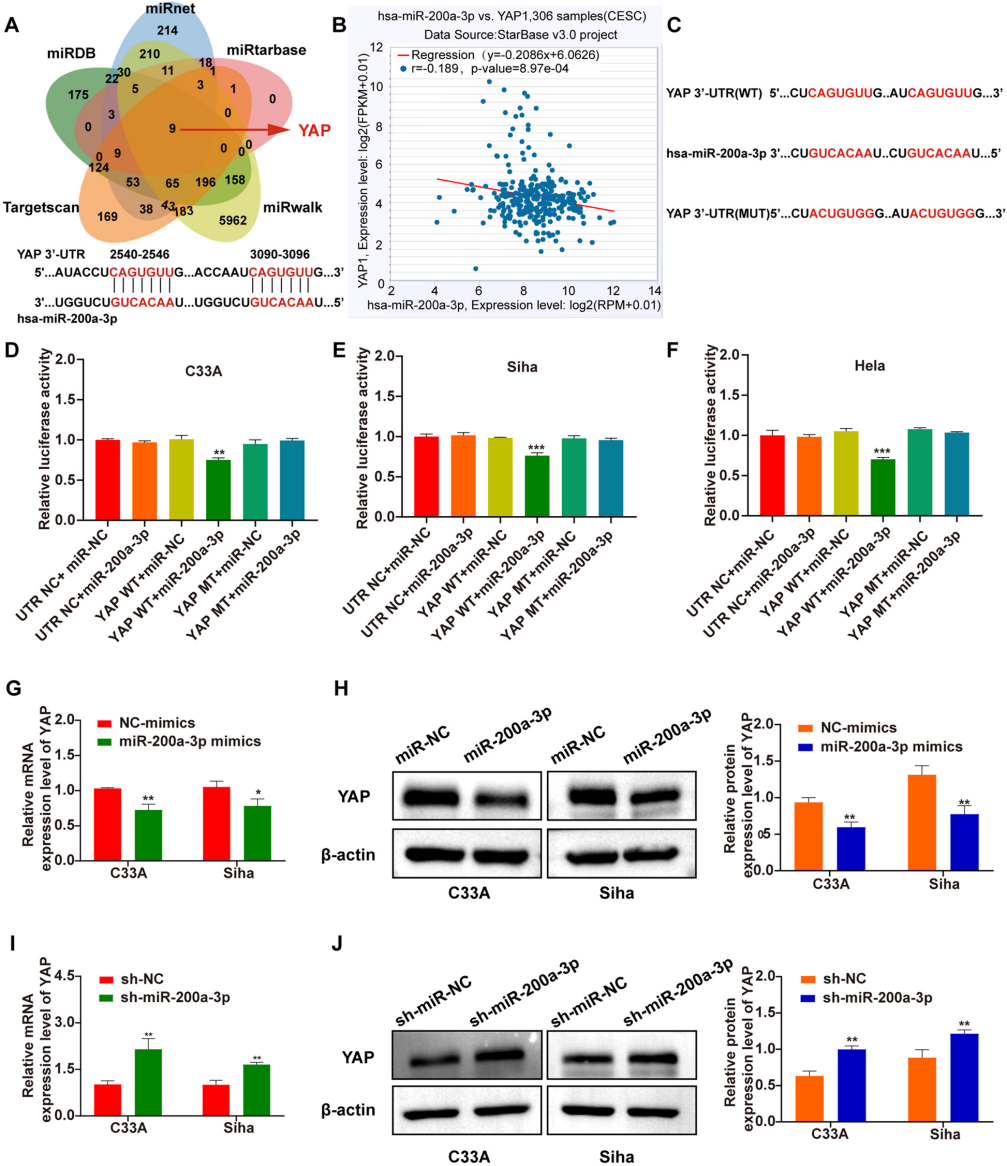
Effects of miR-200a-3p on function of YAP are differentially related to HPV statuses. Intersection Venn diagram for potential downstream targets of miR-200a-3p delivered from 5 bioinformatics Databases (up), and schematic depiction of the double-strand formation of miR-200a-3p with the 3’-UTR of YAP (below) (**A**). Negative correlation between miR-200a-3p and YAP expression was analyzed through the starBase 3.0 (**B**). Schematic diagrams of human YAP 3’-UTR luciferase constructs with YAP-3’UTR and miR-200a-3p target sequences **(C)**. Relative luciferase activities in C33A (**D**), Siha **(E)** and Hela (**F**) with co-transfection of YAP and miR-200a-3p, respectively. Significant decreases in levels of YAP mRNA **(G)** and YAP protein (**H**) in C33A and Siha cells following up-regulation of miR-200a-3p, respectively. Significant increases in levels of YAP mRNA (**I**) and YAP protein (**J**) in C33A and Siha cells following knockdown of miR-200a-3p, respectively. *p < 0.05, **p < 0.01, ***p < 0.001, ns: not significant.

YAP, as the downstream target of and its binding site with miR-200a-3p, was predicted through Targetscan (Fig. 2A), and the data from starBase3.0 [18] showed a negative correlation between YAP and miR-200a-3p in cervical neoplasm tissues (Fig. 2B). These bioinformatic findings were then validated using the YAP 3’-UTR-WT/MUT plasmid and miR-200a-3p/miR-NC luciferase reporter systems (Fig. 2C). The relative luciferase activities of YAP and miR-200a-3p were significantly decreased, indicating that two genes were directly bound in three cell lines (Fig. 2D-F), respectively. Overexpression of miR-200a-3p resulted in down-regulation of YAP expression (Fig. 2G, H), whereas knockdown of miR-200a-3p (Supplementary Fig. S1B in Additional file 1) led to elevations of YAP expression, in HPV-negative C33A and HPV16-positive Siha (Fig. 2I, J), but not in HPV18-positive Hela cells (Supplementary Fig. S2 A-D in Additional file 1).

### Up-regulation of YAP promotes growth and metastasis in the CC cells unrelated to HPV statuses

**Fig. 3:**
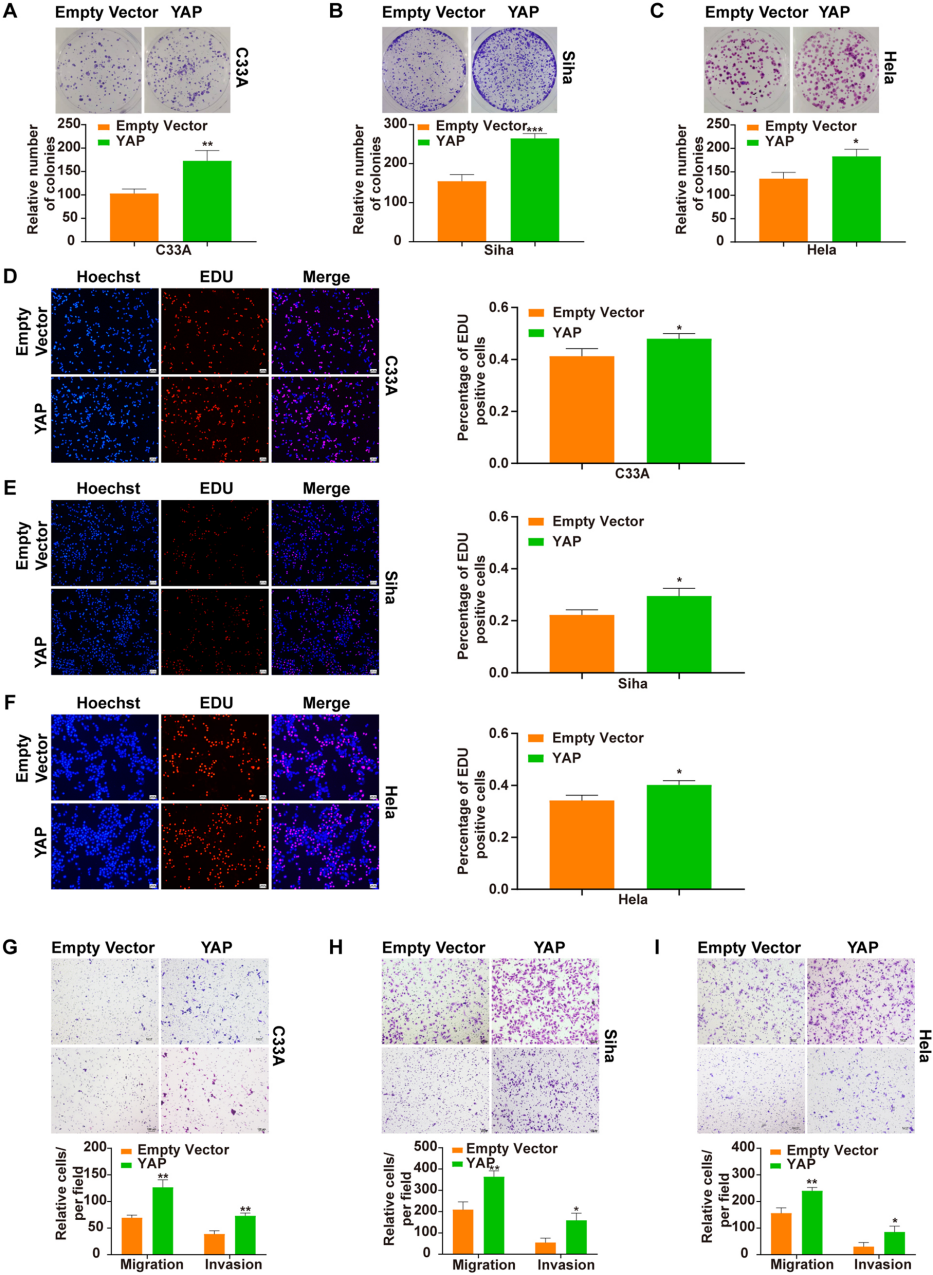
Effects of YAP overexpression on proliferation and invasion of CC cells unrelated to HPV statuses. Colony forming was promoted in C33A (**A**), Siha (**B**) and Hela cells (**C**). EDU-positive cells were increased in C33A (**D**), Siha (**E**) and Hela cells (**F**). Cell migration and invasion were enhanced in C33A (**G**), Siha (**H**) and Hela cells (**I**), respectively. 100 × Magnification. *p < 0.05, **p < 0.01, ***p < 0.001, ns: not significant

With overexpression of YAP in the three cell lines (Supplementary Fig. S1C in Additional file 1), respectively, the colony formation, EDU and transwell assays were performed and showed that YAP overexpression promoted the cell growth, migration and invasion in HPV-negative C33A cells (Fig. 3A, D and G), HPV16-positive Siha (Fig. 3B, E and H) and HPV18-positive Hela cells (Fig. 3C, F and I).

### Differential responses of miR-200a-3p-YAP signal transduction to HPV-negative C33A and HPV16-positive Siha cells

**Fig. 4:**
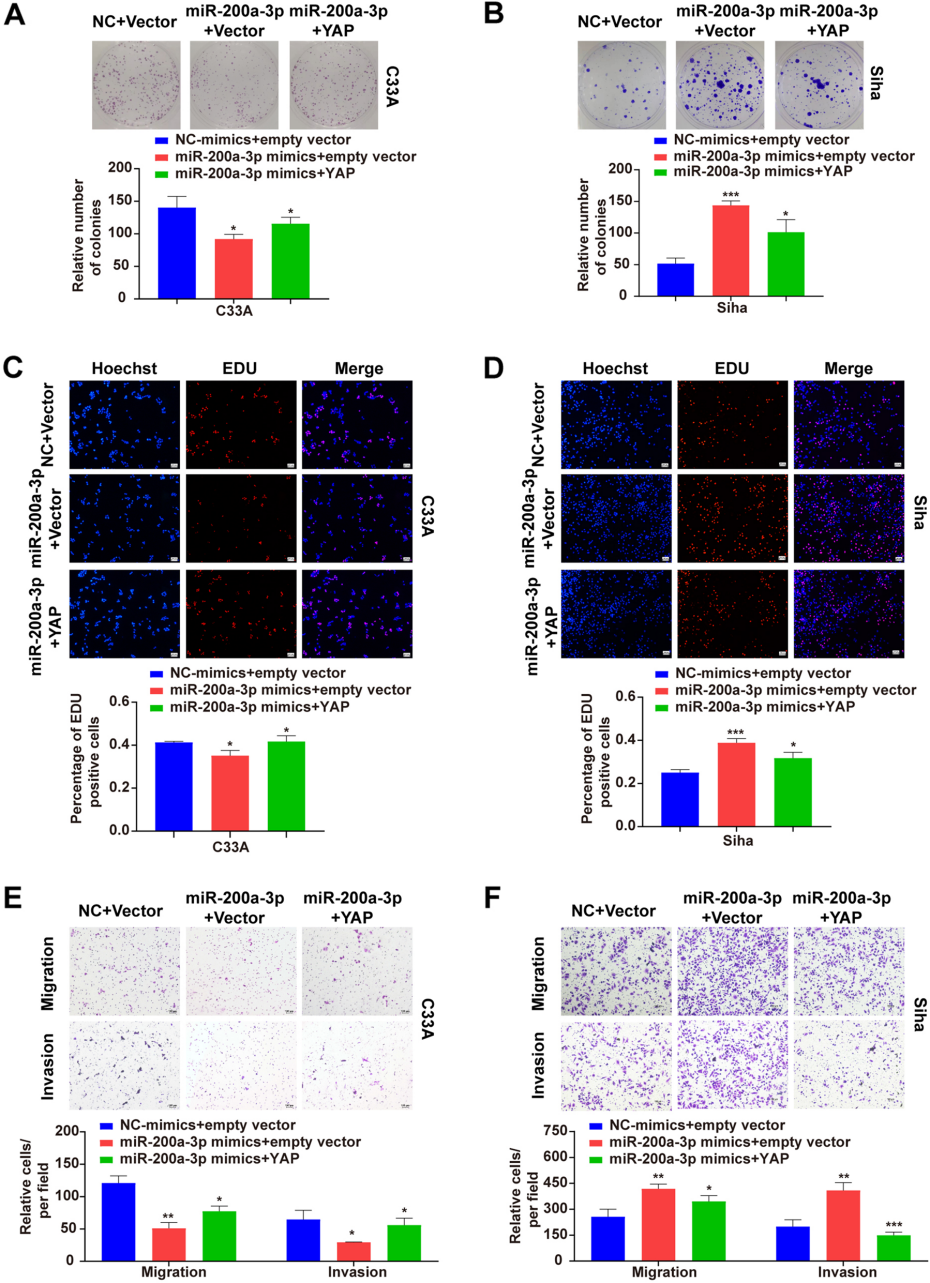
Differential responses of miR-200a-3p-YAP signal transduction to HPV-negative C33A and HPV16-positive Siha cells. Colony forming was promoted in C33A (**A**) but inhibited in Siha (**B**) with co-up-regulation of YAP and miR-200a-3p, respectively. EDU-positive cells were increased in C33A (**C**) but decreased in Siha (**D**) with co-up-regulation of YAP and miR-200a-3p, respectively. Cell migration and invasion were enhanced in C33A (**E**) but declined in Siha (**F**) with co-up-regulation of YAP and miR-200a-3p, respectively. 100 × Magnification. *p < 0.05, **p < 0.01, ***p < 0.001, ns: not significant

Co-up-regulation of miR-200a-3p and YAP in C33A and Siha cells were performed to further examine the effects of miR-200a-3p-YAP signal transduction on the biological changes in HPV-negative and HPV16-positive cells. The data from the colony formation and EDU assays showed that the miR-200a-3p-mediated inhibitory effects in HPV-negative C33A cells (Fig. 4A, C and E) or the promoting effects in HPV16-positive Siha cells (Fig. 4B, D and F) on the cell proliferation, migration and invasion were accordingly counteracted by YAP overexpression.

### Preliminary investigation into the transcriptional changes of miR-200a-3p and YAP in clinical cervical mucus samples

**Fig. 5:**
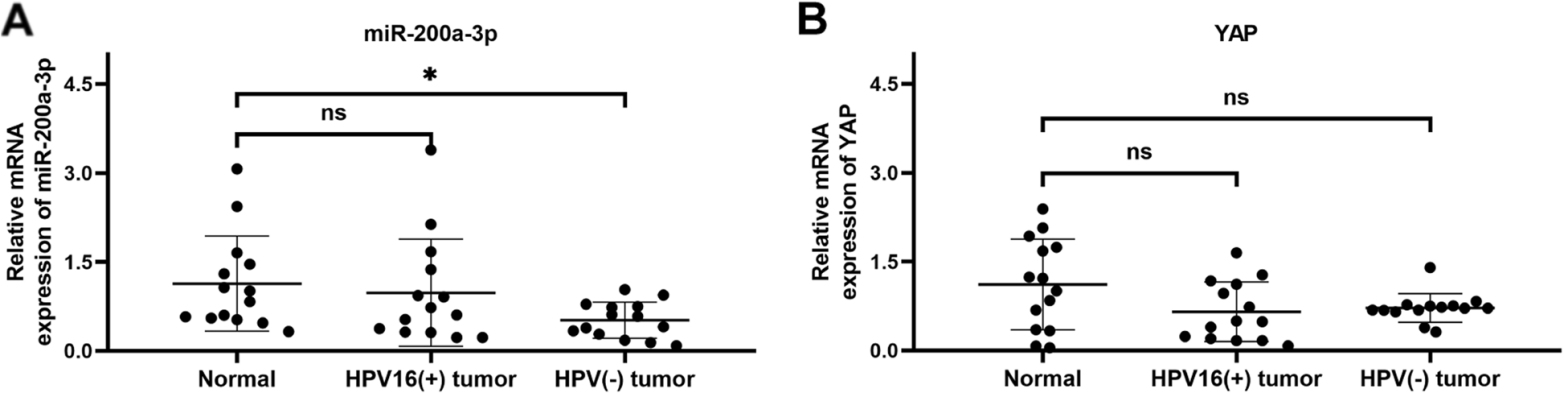
The transcriptional levels of miR-200a-3p and YAP in clinical cervical mucus samples**.** Levels of miR-200a-3p mRNA (**A**) and YAP protein (**B**) in normal cervical mucus (*n* = 14), HPV16-positive (*n* = 14) and HPV-negative cervical neoplasm mucus (*n* = 14). *p < 0.05, **p < 0.01, ***p < 0.001, ns: not significant

The mucus samples were divided into three groups (Normal group, HPV16( +) tumor group, HPV (-) tumor group) according to the results of HPV genotyping and routine pathological diagnosis. In HPV16( +) tumor group, there were 12 patients with HPV16( +) only and 2 with HPV16( +)/53( +). The mRNA expression levels of miR-200a-3p and YAP in the cervical mucus samples from the HPV-negative and HPV16-positive CC patients were examined via qRT-PCR. The results showed that the mucus miR-200a-3p was down-regulated in the HPV-negative CC patients, while no significant changes were observed in the HPV16-positive patients (Fig. 5A). No functional changes in the cervical mucus YAP were found in the HPV-negative and HPV16-positive CC patients compared to the control (Fig. 5B).

## Discussion

miR-200a-3p possesses important regulatory functions differentially in different cancers such as lung, colorectal and breast cancers [19–21], suggesting that miR-200a possesses dual functions, i.e., as an oncogene or as a tumor suppressor, dependent of the differences in cancer types and the tumorous microenvironments. Cervical cancer seems pathogenetically to be far more complex than other kinds of cancers largely due to its association with HPV. Although miR-200a-3p has been revealed to have potential value for predicting CC patient survival [22], the differential cellular and molecular mechanisms underlying miR-200a-3p-mediated growth and metastasis of HPV-negative and HPV-positive CC cells have not been explored.

The data from the present study showed that up-regulation of miR-200a-3p inhibited the cell growth, migration and invasion in HPV-negative C33A cells but promoted the growth and metastasis in HPV-positive Siha and Hela cells (Fig. 1), indicating that miR-200a-3p exerted a dual regulatory role differentially in HPV-negative and HPV-positive CC cells. This result was supportive of a previous study, in which miR-9-5p was shown to play a dual role in CC in histological type- and HPV type-dependent manners [23]. All these findings suggest that the pathological development of CC might not be driven exclusively by HPV but could also be induced by other molecular and cellular events independent of HPV.

By means of the bioinformatics analysis and the luciferase reporter assays, we further verified that YAP appeared to be a direct target of miR-200a-3p not only in C33A but in Siha and Hela cells as well (Fig. 2A, F). Indeed, the expression levels of YAP were decreased or increased accordingly only in HPV-negative C33A and HPV16-positive Siha cells (Fig. 2G, H, I and J) but not in HPV18-positive Hela cells (Supplementary Fig. S2 in Additional file 1), following up- or down-regulation of miR-200a-3p, suggesting that miR-200a-3p-mediated functional changes of YAP in CC cells might be differed from existence or inexistence of HPV and even from different subtypes of HPV.

In addition, the data showed that up-regulation of YAP enhanced the cell growth and metastasis not only in C33A but also in Siha and Hela cells (Fig. 3), which agreed with findings by others [13, 14, 17, 24] and suggesting that the functional changes of YAP might be responsible for the biological alterations in these CC cells seemingly unrelated to HPV.

Given that different results in C33A and Siha cells described above, interestingly, following up-regulation of miR-200a-3p, overexpression of YAP exhibited a counteraction against miR-200a-3p-mediated inhibitory effects in HPV-negative C33A cells (Fig. 4A, C and E) or the promoting effects in HPV16-positive Siha cells (Fig. 4B, D and F). This finding appears to be fundamentally important for better understanding of biological mechanisms of CC, and it also provides experimental evidence with clinical implications that miR-200a-3p-YAP signal transduction would result in regulatory consequences differentially in HPV-negative or HPV-positive CC cells. It is also worth to concern more about the miR-200a-3p-mediated changes of YAP in Siha cells (Fig. 4B, D and F). One possible explanation could be that YAP could function not merely as the downstream target of miR-200a-3p, but also be modulated by other signals within the tumorous microenvironment in HPV16-positive Siha cells. Indeed, previous studies demonstrated that YAP could inhibit or enhance some miRNAs biogenesis in a cell density-dependent manner [12], and that YAP and miR-130a-VGLI4 could form a positive feedback loop that promoted cell growth and tumorigenesis [25]. In addition, it was shown that the miR-200 family was closely related to multiple molecules (including LATS1/2, FAT1/2 and PTPN14) of the Hippo signaling pathway [26], and LATS1/2-mediated phosphorylation of YAP or TAZ was involved in the cell growth and apoptosis [27]. However, some studies also found a negative feedback regulation between YAP / TAZ and LATS1/2, in which significant up-regulation and elevated phosphorylation of LATS2 were observed following YAP overexpression, and LATS2 instead exhibited promoting effects on phosphorylation and degradation of YAP [28–30]. Nevertheless, in the present study, if the interaction of miR-200a-3p with YAP and its upstream inhibitory kinases of Hippo signaling pathway (especially LATS2) would also be likely to have feedback signaling and hereby to govern cells’ fate in Siha cells needs to be investigated further.

In addition, several studies found that both YAP and TAZ co-activation could build a negative feedback loop of the Hippo pathway or regulate microRNA biogenesis through let-7 [28, 31], and YAP/TAZ combined with other molecules were involved in the occurrence and progression of various cancers [32–34]. Overexpression of TAZ could exert promoting effects on the tumorigenicity of CC cells, but one study revealed TAZ could be negatively regulated by YAP abundance in mammal cells [35, 36]. Therefore, there was a possibility that TAZ could be involved in the interaction of miR-200a-3p and YAP.

It is reported that miRNAs not only existed in tissues, but also in body fluids such as serum, urine and so on [37]. A signature including several miRNAs in urine was examined since bladder tumors are in direct contact with urine, which suggested it is the ideal body fluid for bladder tumors [38], and one study has revealed that four miRNAs in cervical mucus were defined as the promising biomarkers for CC and high-grade CINs [39], so we assumed that cervical mucus was an ideal material for cervical neoplasm for the same reason. Therefore, as an initial attempt to validate the in vitro findings described above, the transcriptional levels of miR-200a-3p and YAP in the cervical mucus samples from HPV-negative and HPV16-positive CC patients were determined, respectively. MiR-200a-3p expression was decreased significantly in the HPV-negative CC group, while unchanged in the HPV16-positive CC patients (Fig. 5A). Beyond our expectation, no difference in YAP expression was observed amongst these groups (Fig. 5B). Apparently, it was difficult to make a meaningful comparison of these results with the in vitro findings at the current stage of this study. Nevertheless, a limitation in terms of relatively fewer testing samples that might result in uncertain data should be considered, more clinical data obtained from clinical cervical tissues are necessary in the future.

In summary, this study demonstrates that either miR-200a-3p or YAP could differentially regulate the growth and metastasis of CC cells in an HPV-related manner, and the miR-200a-3p-mediated functional changes of YAP also exhibited regulatory effects on the tumorous cells’ fate differing from HPV-negative C33A and HPV16-positive Siha cells. The differential regulation may be affected not only by presence or absence of HPV but even by the different subtypes of HPV or the influence of microenvironment of CC cells as well. Our findings revealed a novel miR-200a-3p-YAP signaling axis in HPV-negative C33A cells, and this signaling axis may have potential values in the treatment and prognosis specifically for HPV-negative patients with cervical cancer. Taking together, the findings may also have fundamental implications in giving explanations, at least partially, for the occurrence and development of cervical cancer not only in HPV-positive but particularly also in HPV-negative patients.

## Conclusion

In conclusion, this study suggests that miR-200a-3p can differentially regulate the proliferation and metastasis of cervical cancer cells in an HPV-related manner, and miR-200a-3p-mediated functional changes of YAP also exert differential effects on the tumorous cells’ fate in HPV-negative and HPV-positive cervical cancer.

## Supplementary Information

Below is the link to the electronic supplementary material.

**Additional file 1**: **Supplementary Fig. S1.** qRT-PCRassessmentsoftransfectionefficiencies The transfection efficiencies of miR-200a-3p mimics (**A**), sh-miR-200a-3p (**B**), and YAP(**C**)wereperformedinC33A,SihaandHelacells.*p<0.05, **p<0.01,***p<0.001,ns:notsignificant. The authors declare that they have no competing interests. **Supplementary Fig. S2.** themRNAandproteinlevelsofYAPinHelacells Significant decreases in levels of YAP mRNA (**A**) and YAP protein (**B**) following up-regulations of miR-200a-3p, or significant increases in levels of YAP mRNA (**C**) and protein (**D**) following down-regulations of miR-200a-3p were not observed in Helacells.*p<0.05,**p<0.01,***p<0.001,ns:notsignificant.

**Additional file 2:** **Fig. 2H.** Full-length blots in Figure 2H C33A (left) and Siha (right) cells. The red solid lines are the figures in the manuscript. **Fig. 2J. **Full-length blots in Figure 2J C33A (left) and Siha (right) cells. The red solid lines are the figures in the manuscript**. Supplementary Fig. S2. **Full-length blots in Supplementary Fig S2. The red solid lines are the figures in the manuscript.

**Additional file 3.**

## Data Availability

All data analyzed during this study can be found in below websites. Prediction of potential ta-rgets of miRNA was performed by using internet databases including miRWalk (http://mirwalk.umm.uni-heidelberg.de), miRNet (https://www.mirnet.ca/miRNet/home.xhtml), miRDB(http://mirdb.org/download.html), Targetscan (http://www.targetscan.org/vert_72/) and miRTarBase ( data was presented in Additional file 3). Data obtained from Targetscan (http://www.targetscan.org/vert_72/) was used to predict the binding sites. The data from Starbase 3.0 (https://starbase.sysu.edu.cn/) was used to show the correlation between miRNA and target genes.

## Abbreviations

CC: Cervical cancer
FBS: Fetal bovine serum
HPV: Human papillomavirus
miRNAs: MicroRNAs
MUT: Mutation
NC: Negative control
PAGE: Polyacrylamide gel electrophoresis
PVDF: Polyvinylidene Fluoride
qRT-PCR: Quantitative Real-time PCR
RIPA: Radio Immunoprecipitation Assay
RNA: Ribonucleic Acid
SDS: Sodium Dodecyl Sulfate
WT: Wild type
YAP: Yes-associated protein
3’-UTR: 3’-Untranslated regions
