# Supplementary material for: Interaction of miR-200a-3p with YAP regulates cell proliferation and metastasis differentially in HPV-positive and HPV-negative cervical cancer cells
Source: BMC Cancer. 2022 Oct 4;22:1039. doi: 10.1186/s12885-022-10118-0 (PMC9533500; doi:10.1186/s12885-022-10118-0)

Supplementary Fig. S1 qRT-PCR assessments of transfection efficiencies

The transfection efficiencies of miR-200a-3p mimics **(A)**, sh-miR-200a-3p **(B)**, and YAP **(C)** were performed in C33A, Siha and Hela cells. *$p < 0.05$, **$p < 0.01$, ***$p < 0.001$, ns: not significant.

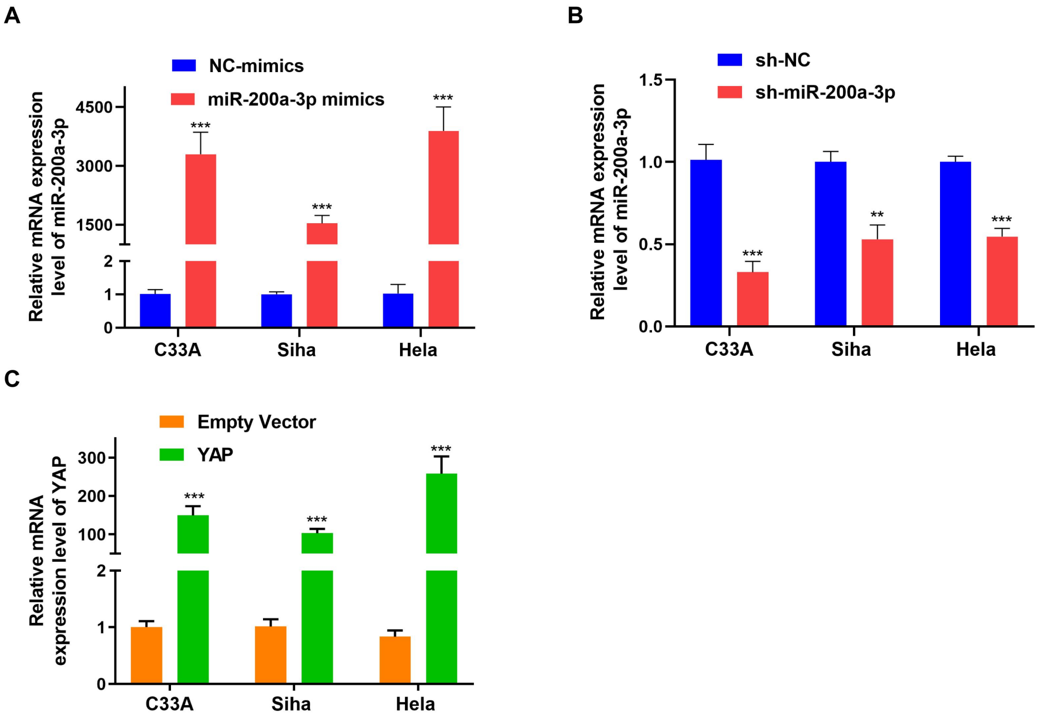

Supplementary Fig. S2 the mRNA and protein levels of YAP in Hela cells

Significant decreases in levels of YAP mRNA **(A)** and YAP protein **(B)** following up-regulations of miR-200a-3p, or significant increases in levels of YAP mRNA **(C)** and protein **(D)** following down-regulations of miR-200a-3p were not observed in Hela cells. *$p < 0.05$, **$p < 0.01$, ***$p < 0.001$, ns: not significant.

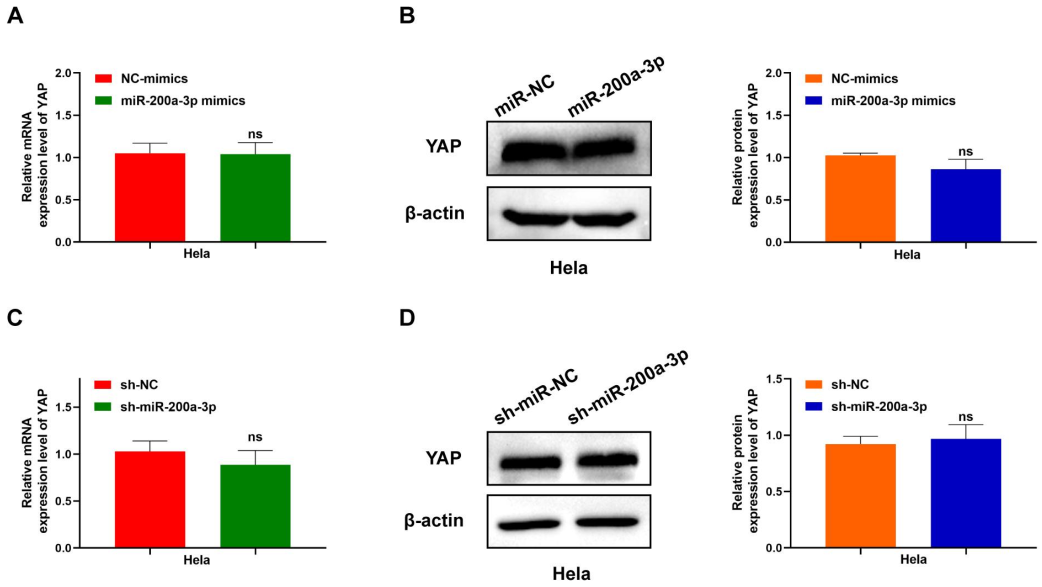

Supplement: Supplementary file 1 — Additional file 1: Supplementary Fig. S1. qRT-PCRassessmentsoftransfectionefficiencies The transfection efficiencies of miR-200a-3p mimics (A), sh-miR-200a-3p (B), and YAP(C)wereperformedinC33A,SihaandHelacells.*p<0.05, **p<0.01,***p<0.001,ns:notsignificant. The authors declare that they have no competing interests. Supplementary Fig. S2. themRNAandproteinlevelsofYAPinHelacells Significant decreases in levels of YAP mRNA (A) and YAP protein (B) following up-regulations of miR-200a-3p, or significant increases in levels of YAP mRNA (C) and protein (D) following down-regulations of miR-200a-3p were not observed in Helacells.*p<0.05,**p<0.01,***p<0.001,ns:notsignificant. [file 12885_2022_10118_MOESM1_ESM.pdf]
