# Supplementary material for: Interaction of miR-200a-3p with YAP regulates cell proliferation and metastasis differentially in HPV-positive and HPV-negative cervical cancer cells
Source: BMC Cancer. 2022 Oct 4;22:1039. doi: 10.1186/s12885-022-10118-0 (PMC9533500; doi:10.1186/s12885-022-10118-0)

Fig. 2H Full-length blots in Figure 2H C33A (left) and Siha (right) cells. The red solid lines are the figures in the manuscript.

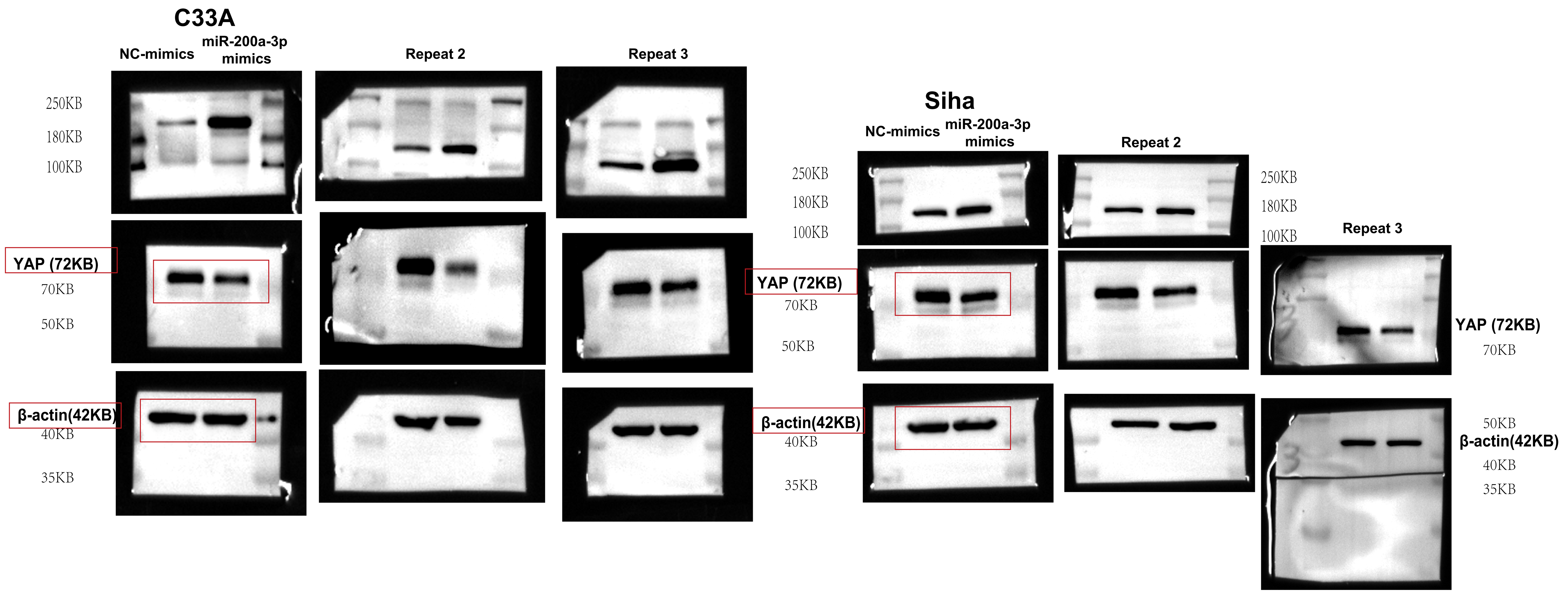

Fig. 2J Full-length blots in Figure 2J C33A (left) and Siha (right) cells. The red solid lines are the figures in the manuscript.

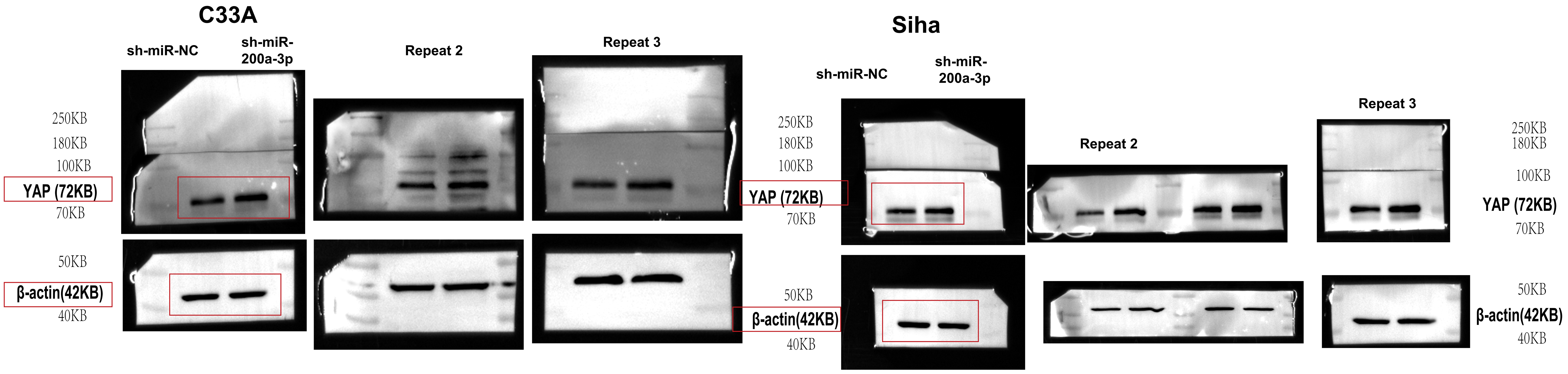

Supplementary Fig. S2 Full-length blots in Supplementary Fig S2. The red solid lines are the figures in the manuscript.

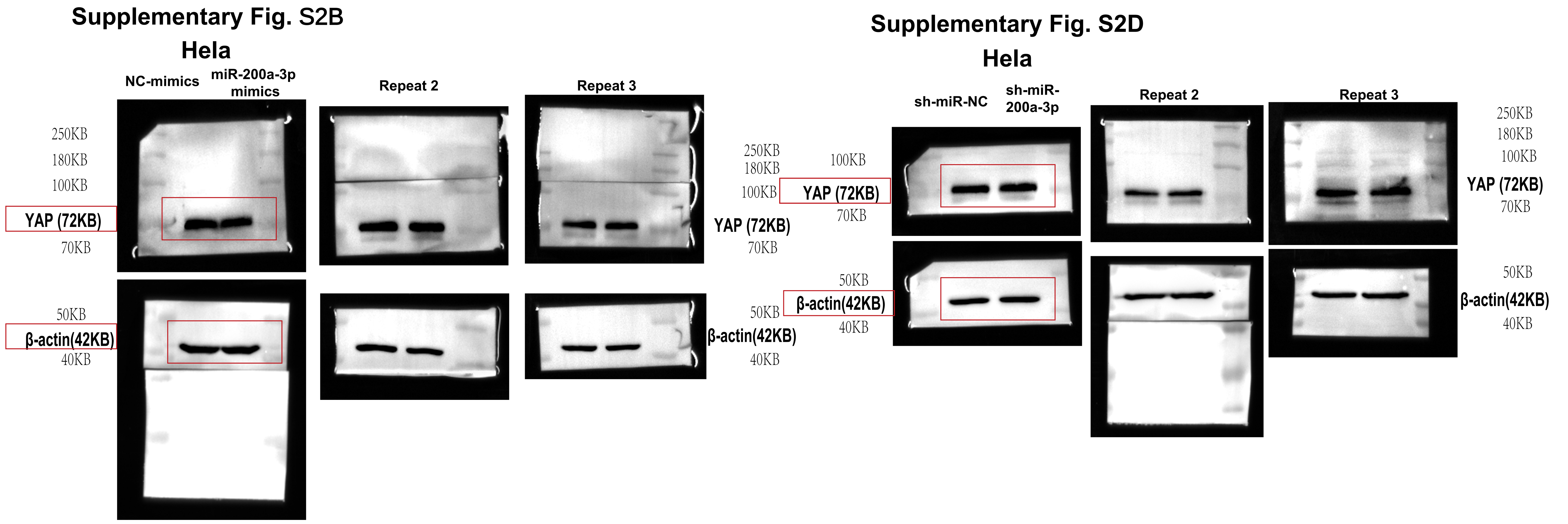

Supplement: Supplementary file 2 — Additional file 2: Fig. 2H. Full-length blots in Figure 2H C33A (left) and Siha (right) cells. The red solid lines are the figures in the manuscript. Fig. 2J. Full-length blots in Figure 2J C33A (left) and Siha (right) cells. The red solid lines are the figures in the manuscript. Supplementary Fig. S2. Full-length blots in Supplementary Fig S2. The red solid lines are the figures in the manuscript. [file 12885_2022_10118_MOESM2_ESM.pdf]
